# Simple demographic characteristics and laboratory findings on admission may predict in-hospital mortality in patients with SARS-CoV-2 infection: development and validation of the covid-19 score

**DOI:** 10.1186/s12879-021-06645-z

**Published:** 2021-09-14

**Authors:** Marta Obremska, Monika Pazgan-Simon, Katarzyna Budrewicz, Lukasz Bilaszewski, Joanna Wizowska, Dariusz Jagielski, Beata Jankowska-Polanska, Klaudiusz Nadolny, Jarosław Madowicz, Jolanta Zuwala-Jagiello, Dorota Zysko, Waldemar Banasiak, Krzysztof Simon

**Affiliations:** 1 Department of Preclinical Research, Wroclaw Medical University, Wroclaw, Poland; 2 Ist Department of Infectious Diseases Regional Specialistic Hospital, Wroclaw, Poland; 3 Department of Emergency Medicine, Wroclaw Medical University, ul. Borowska 213, 50-556 Wroclaw, Poland; 4 Centre for Heart Diseases, 4th Military Hospital, Wroclaw, Poland; 5 Department of Clinical Nursing, Wroclaw Medical University, Wroclaw, Poland; 6 Department of Emergency Medical Service, Higher School of Strategic Planning in Dabrowa Gornicza, Dabrowa Gornicza, Poland; 7 Faculty of Medicine, Katowice School of Technology, Katowice, Poland; 8 Provincial Specialist Hospital, Tychy, Poland; 9 Department of Health Sciences, Higher School of Strategic Planning in Dabrowa Gornicza, Dabrowa Gornicza, Poland; 10 Department of Pharmaceutical Biochemistry, Wroclaw Medical University, Wroclaw, Poland; 11 Department of Infectious Diseases and Hepatology, Wroclaw Medical University, Wroclaw, Poland

## Abstract

**Background:**

Coronavirus disease 2019 (COVID-19) caused by severe acute respiratory syndrome coronavirus 2 (SARS-CoV-2) constitutes a major health burden worldwide due to high mortality rates and hospital bed shortages. SARS-CoV-2 infection is associated with several laboratory abnormalities. We aimed to develop and validate a risk score based on simple demographic and laboratory data that could be used on admission in patients with SARS-CoV-2 infection to predict in-hospital mortality.

**Methods:**

Three cohorts of patients from different hospitals were studied consecutively (developing, validation, and prospective cohorts). The following demographic and laboratory data were obtained from medical records: sex, age, hemoglobin, mean corpuscular volume (MCV), platelets, leukocytes, sodium, potassium, creatinine, and C-reactive protein (CRP). For each variable, classification and regression tree analysis were used to establish the cut-off point(s) associated with in-hospital mortality outcome based on data from developing cohort and before they were used for analysis in the validation and prospective cohort. The covid-19 score was calculated as a sum of cut-off points associated with mortality outcome.

**Results:**

The developing, validation, and prospective cohorts included 129, 239, and 497 patients, respectively (median age, 71, 67, and 70 years, respectively). The following cut of points associated with in-hospital mortality: age > 56 years, male sex, hemoglobin < 10.55 g/dL, MCV > 92.9 fL, leukocyte count > 9.635 or < 2.64 10^3^/µL, platelet count, < 81.49 or > 315.5 10^3^/µL, CRP > 51.14 mg/dL, creatinine > 1.115 mg/dL, sodium < 134.7 or > 145.4 mEq/L, and potassium < 3.65 or > 6.255 mEq/L. The AUC of the covid-19 score for predicting in-hospital mortality was 0.89 (0.84–0.95), 0.850 (0.75–0.88), and 0.773 (0.731–0.816) in the developing, validation, and prospective cohorts, respectively *(P* < 0.001The mortality of the prospective cohort stratified on the basis of the covid-19 score was as follows: 0–2 points,4.2%; 3 points, 15%; 4 points, 29%; 5 points, 38.2%; 6 and more points, 60%.

**Conclusion:**

The covid-19 score based on simple demographic and laboratory parameters may become an easy-to-use, widely accessible, and objective tool for predicting mortality in hospitalized patients with SARS-CoV-2 infection.

## Background

Coronavirus disease 2019 (COVID-19) is a current global pandemic caused by severe acute respiratory syndrome coronavirus 2 (SARS-CoV-2) [1]. The clinical spectrum of COVID-19 ranges from asymptomatic infection to a severe or even fatal disease in some cases.COVID-19 poses an enormous challenge to healthcare systems. The hospitalization rate is high, and there is a significant shortage of intensive care unit (ICU) beds in some countries [2]. The early stratification of mortality risk may facilitate decision-making about hospitalization and referral to the ICU. Clinical evaluation of the patient on hospital admission, often involving collection of demographic data as well as laboratory and radiologic findings, is one of the methods of risk stratification [3–5]. However, the evaluation of clinical data is susceptible to observer bias. Moreover, in real-life conditions, some data may be overlooked due to excessive workload of the medical personnel [6]. On the other hand, laboratory tests are less prone to bias. They provide objective results that allow clinicians to make quick decisions about patient care. As such, they play an important role in the decision-making process in almost all diseases.

The aim of the study was to develop and validate a simple scoring system (covid-19 score) based on demographic data and routine laboratory measurements on admission to predict the risk of death in hospitalized patients with COVID-19. The covid-19 score using simple observer-independent parameters might facilitate the stratification of patients with COVID-19 who are at increased risk of in-hospital death. Moreover, it might be useful for identifying homogeneous groups of patients for efficiency comparisons between different healthcare systems.

## Methods

### Participants

Study participants came from 3 different hospitals in Poland. The developing cohort consisted of 129 patients from County Hospital in Boleslawiec, treated between March and July 2020. The validation cohort included 239 patients from Regional Specialist Hospital in Wroclaw, treated between June and August 15, 2020. Finally, the prospective cohort consisted of 497 patients from the 4th Military Hospital in Wroclaw, treated between October and December 2020.

Electronic medical records were searched to retrieve demographic data (age and sex) as well as laboratory findings on admission (hemoglobin, creatinine, potassium, sodium, and C-reactive protein [CRP] levels, mean corpuscular volume (MCV), platelet and leukocyte count), as well as the mode of discharge.

The inclusion criterion was the diagnosis of SARS-CoV-2 infection confirmed by a reverse transcriptase-polymerase chain reaction test of a nasopharyngeal swab. The exclusion criteria were as follows: lack of any of the studied laboratory parameters, patient transfer to another hospital, discharge against medical advice, or leaving the hospital without being discharged*.*

### Risk assessment tool

A classification and regression tree (CART) analysis was used to establish the cut-off points for each variable that were associated with mortality outcome. The CART analysis provides rules that predict an outcome variable from explanatory variables. For each variable, we selected a cut-off point which was a split criterion that divided the developing cohort into the most homogenous subgroups in terms of in-hospital survival. The analysis was performed separately for each independent variable. The first split criterion was used as a cut-off point. However, for laboratory variables expected to have a poor prognosis of the extreme values of the range, the second split criterion was also chosen as the cut-off point. Each variable that was within the threshold associated with mortality outcome was assigned 1 point. The covid-19 score was calculated as the sum of points for each analyzed parameter. Global Cross validation cost and its standard deviation were calculated.

The area under the receiver operating characteristic curve (AUC) of the covid-19 score for predicting in-hospital mortality was calculated in the developing, validation, and prospective cohorts.

The cut-off points used for generating the score were established after obtaining data from the developing cohort and before they were used for analysis in the validation and prospective cohort. The outcome was in-hospital mortality.

The study was approved by Bioethics Committee of Wroclaw Medical University (No/ 275/2020). The routine data to develop the prediction model was collected retrospectively; therefore, written informed consent to participate in the study was not required. The Bioethics Committee approved the publication of anonymized data.

### Statistical analysis

Continuous variables were presented as means and standard deviations or medians and interquartile ranges according to their distribution, and were compared with the Student’s *t*-test or the Mann–Whitney *U* test. Discrete variables were presented as numbers and percentages and compared with the chi-squared test. The CART analysis was used to identify the cut-off points to differentiate between survivors and nonsurvivors, and one point was assigned for each variable that was associated with mortality outcome. The receiver operating characteristic (ROC) curve analysis was used to validate the prediction model. A *P* value of less than 0.05 was regarded as significant.

Statistical analysis was performed using the Statistica 13.3 software (TIBCO Software Inc., Palo Alto, California, United States).

## Results

We collected demographic and laboratory data for developing, validation, and prospective cohorts. The median age of the developing, validation, and prospective cohorts was 71, 67, and 70 years, respectively, and the percentage of male participants was 43%, 56.1%, and 58.8%, respectively. Detailed data are presented in Table 1. The mortality rate was similar in developing and prospective cohort (34.9% vs 29%, *P* = 0.18) but in validation cohort was significantly lower (18.8%) than in developing and prospective cohort ( respectively *P* < 0.001and *P* = 0.004).

**Table 1:** Demographic and laboratory data in the study cohorts

Variable	Developing cohort (n = 129)	Validation cohort (n = 239)	Prospective cohort (n = 497)	*P* value (developing vs validation cohort)	*P* value (developing vs prospective cohort)	*P* value (validation vs prospective cohort)
Age (years), median (IQR)	71 (57–79)	67 (53–95)	70 (56–81)	p < 0.03	0.99	p < 0.004
Male sex, n (%)	56 (43.0)	134 (56.1)	292 (58.8)	0.12	p < 0.02	0.99
Hemoglobin level (g/dL), median (IQR)	12.9 (10.7–14.2)	13.6 (12.4–14.8)	13.2 (11.8–14.5)	p < 0.001	p < 0.048	p < 0.046
Mean corpuscular volume (fL), median (IQR)	87.2 (83.6–91.1)	88.6 (86.2–91.5)	90.2 (86.9–94.0)	p < 0.027	p < 0.001	p < 0.001
Leukocyte count, (10^3^/µL), median (IQR)	6.8 (4.9–9.5)	6.2 (4.8–8.8)	8.1 (5.9–11.3)	0.56	< 0.001	p < 0.001
Platelet count (10^3^/µL), median (IQR)	192 (151–258)	207 (168–259)	215 (167–287)	0.50	0.07	0.99
C-reactive protein (mg/dL), median (IQR)	63.2 (18.5–132.7)	44.8 (10.0–102.1)	48.4 (10.2–123.5)	0.12	0.11	0.99
Creatinine, mg/dL	1.01 (0.76–1.54)	0.90 (0.75–1.12)	1.16 (0.93–1.53)	< 0.001	0.007	0.001
Sodium (mEq/L), median (IQR)	135.7 (132.4–138.7)	140.9 (138.9–142.9)	137 (134–140)	p < 0.001	p < 0.04	p < 0.001
Potassium (mEq/L), median (IQR)	4.1 (3.8–4.5)	4.2 (3.9–4.6)	4.0 (3.7–4.4)	0.85	p < 0.014	p < 0.001

*IQR* interquartile range

**Fig. 1:**
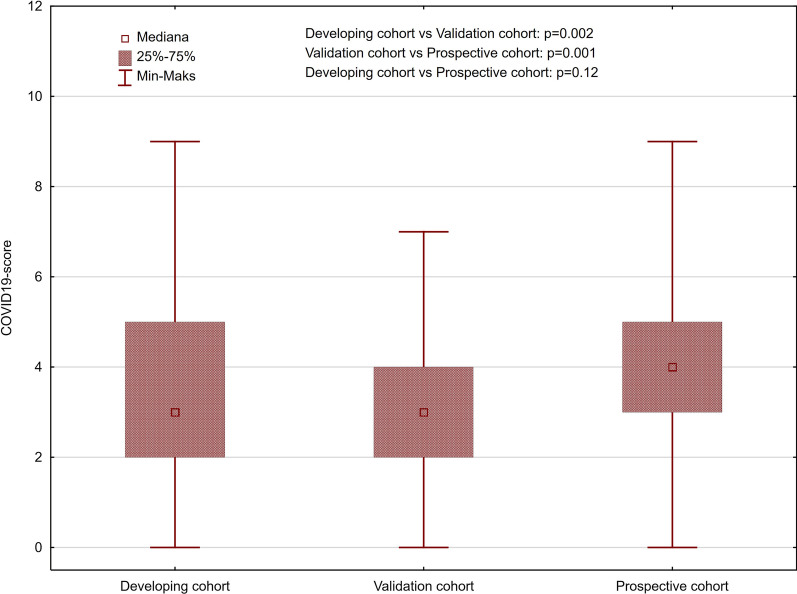
The distribution of covid-19 score in the study cohorts

The cut-off points for predicting in-hospital mortality in the developing cohort obtained from CART analysis are presented in Table 2. The distribution of a covid-19 score in the study cohorts ware presented in Fig. 1.

**Table 2:** Cut-off points for predicting in-hospital mortality: results of the classification and regression tree analysis in developing cohort

Variable	Cut-off point	Global cross-validation cost	SD of the global cross-validation cost
Age (years)	> 56	0.37	0.04
Male sex	Male sex	0.43	0.05
Hemoglobin, g/dL	< 10.55	0.61	0.04
Mean corpuscular volume, fL	> 92.9	0.41	0.04
Leukocyte count, 10^3^/µL	> 9.635 or < 2.64	0.28	0.04
Platelet count, 10^3^/µL	< 81.49 or > 315.5	0.44	0.04
C-reactive protein, mg/dL	> 51.14	0.32	0.04
Creatinine, mg/dL	> 1.115	0.31	0.04
Sodium, mEq/L	< 134.7 or > 145.4	0.51	0.04
Potassium, mEq/L	< 3.65 or > 6.255	0.45	0.05

*SD* standard deviation

**Fig. 2:**
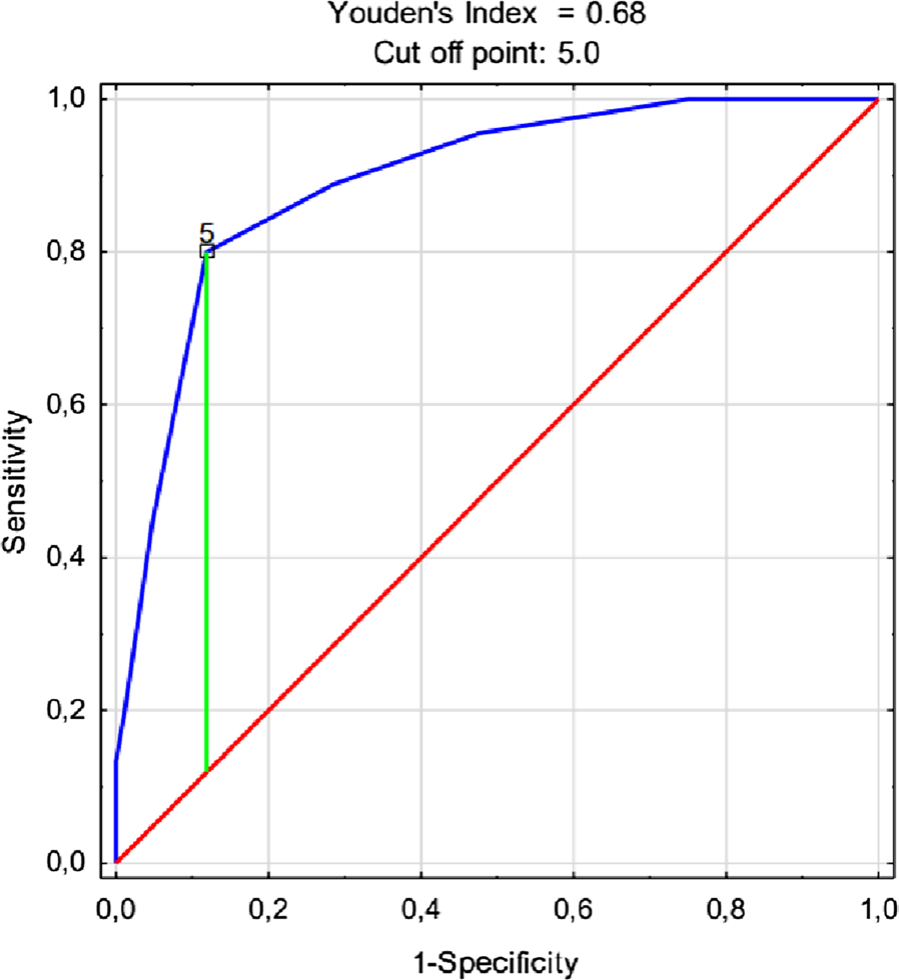
The ROC curve for the prediction of mortality using the covid-19 score in the developing cohort. The AUC of the covid-19 score for predicting in-hospital mortality in developing cohort was 0.89 (0.84–0.95), *P* < 0.001

**Fig. 3:**
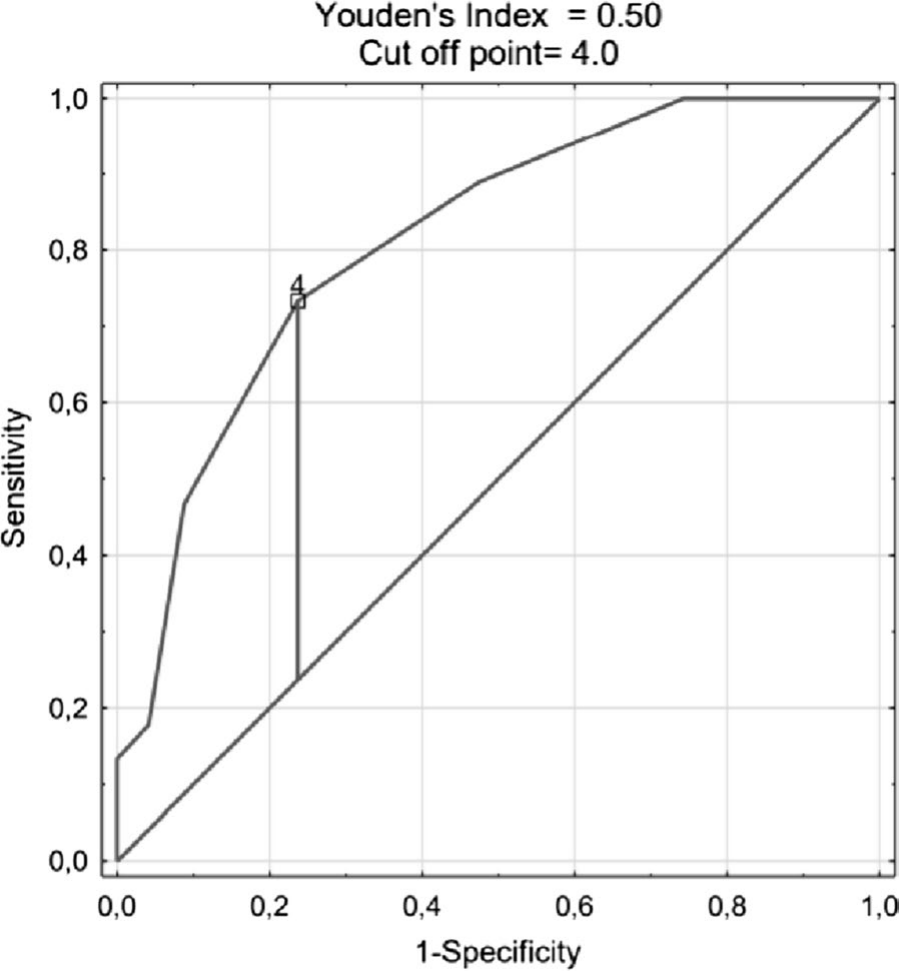
The ROC curve for prediction of mortality using the covid-19 score in the validation cohort. The AUC of the covid-19 score for predicting in-hospital mortality in validation cohort was 0.850 (0.75–0.88), *P* < 0.001

**Fig. 4:**
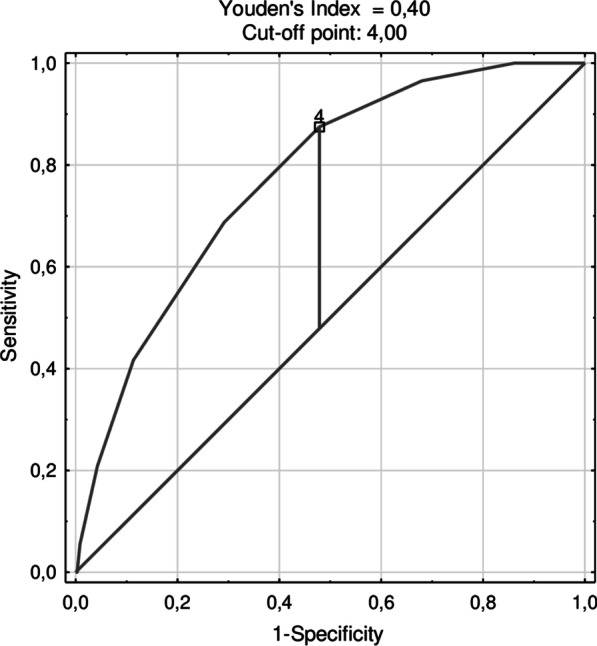
The ROC curve for the prediction of mortality using the covid-19 score in the prospective cohort. The AUC of the covid-19 score for predicting in-hospital mortality in the prospective cohort was 0.773 (0.731–0.816), *P* < 0.001

The ROC curve for the prediction of mortality using the covid-19 score in the developing cohort is presented in Fig. 2. The AUC of the covid-19 -score for predicting in-hospital mortality was 0.89 (0.84–0.95), *P* < 0.001. In the validation cohort, the AUC was 0.850 (0.75–0.88), *P* < 0.001 (Fig. 3). The ROC curve for the prediction of mortality in the prospective cohort is presented in Fig. 4. The AUC of the covid-19 score in the prospective cohort was 0.773 (0.731–0.816), *P* < 0.001.

The distribution of survivors and non-survivors in the prospective cohort according to the covid-19 score is presented in Table 3. The covid-19 score showed higher sensitivity than specificity for predicting in-hospital mortality (Table 3).

**Table 3:** Distribution of survivors and non-survivors in the prospective cohort by the COVID19-score

COVID19-score	No. of participants	No. of non-survivors/survivors	True positive	False positives	False negatives	True negatives	Sensitivity	Specificity
9	1	0/1	0	1	144	352	0.000	0.997
8	10	8/2	8	3	136	350	0.056	0.992
7	34	22/12	30	15	114	338	0.208	0.958
6	55	30/25	60	40	84	313	0.417	0.887
5	102	39/63	99	103	45	250	0.688	0.708
4	93	27/66	126	169	18	184	0.875	0.521
3	84	13/71	139	240	5	113	0.965	0.320
2	69	5/64	144	304	0	49	1.000	0.139
1	37	0/37	144	341	0	12	1.000	0.034
0	12	0/12	144	353	0	0	1.000	0.000

**Fig. 5:**
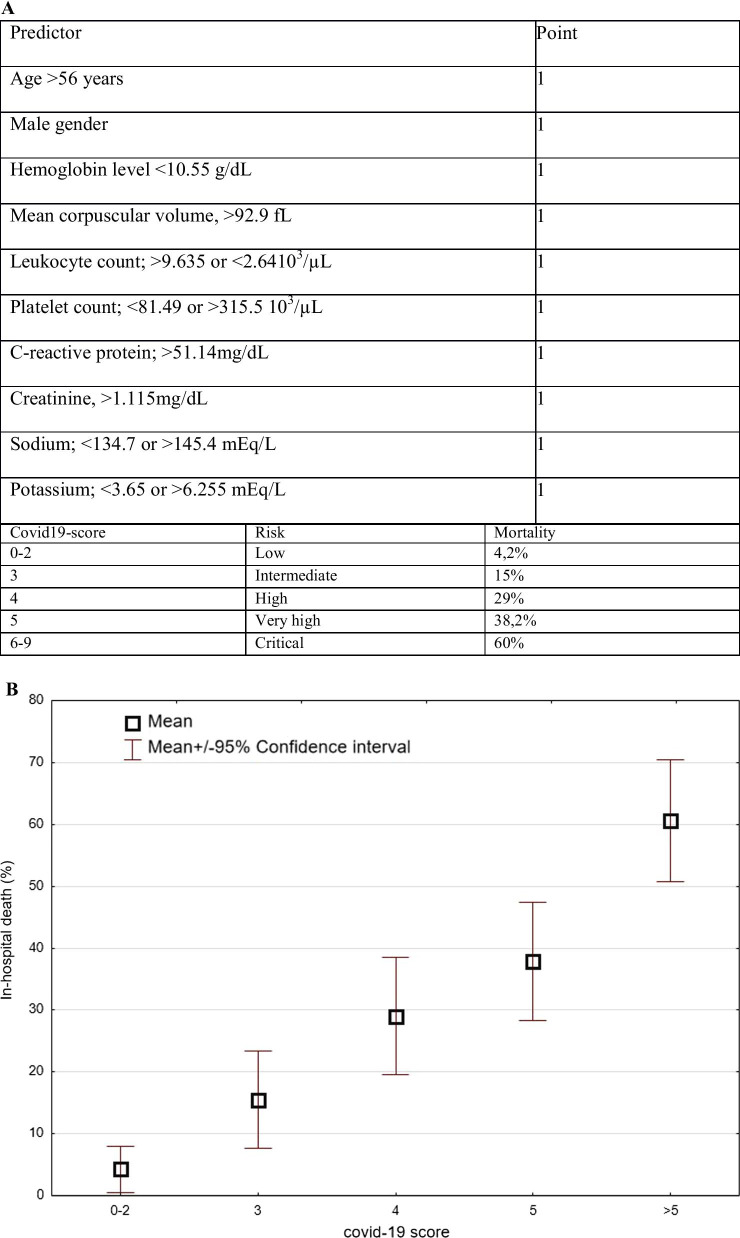
The COVID19-score. **A** Predictors of mortality included in the score with the established cut-off points. The COVID19-score is calculated as a sum of points assigned for each individual parameter according to the established thresholds. The mortality of the prospective cohort stratified on the basis of the COVID19-score. **B** Mortality risk assessment using the COVID19-score

Based on the obtained results, we developed a model to calculate the covid-19 score and predict the risk of in-hospital death in patients with SARS-CoV-2 infection (Fig. 5).

## Discussion

The main finding of this study is that simple demographic characteristics and laboratory findings can be used to predict mortality in hospitalized patients with SARS-CoV-2 infection. Our mortality prediction model is based on objective tests performed on admission. Early evaluation of in-hospital mortality risk is important because it may facilitate the identification of high‐risk individuals.

In the era of the COVID-19 pandemic, numerous models for predicting in-hospital mortality have been developed [7–20]. They are typically based on such data as age, sex, selected aspects of the treatments used, comorbidities, as well as imaging and laboratory findings. However, given the pandemic-related limitations on direct contact with the patient, it may be difficult to obtain a detailed medical history. Comorbidities are common in mortality prediction models. Typically, the severity of a comorbidity is more important than its presence itself. Therefore, we did not include comorbidities in our model because their impact is reflected in laboratory tests. Imaging findings were not included either, because chest computed tomography is not a routine procedure performed on admission in patients with SARS-CoV-19 infection, especially during the pandemic. Oxygen saturation in room air is another important prognostic factor, but in real life, it is difficult to obtain in patients undergoing oxygen therapy. The shortcoming of some prediction scores is limited access to some laboratory tests such as ferritin or interleukin 6. Furthermore, risk scores developed for one population may not be applicable to another because of differences in ethnicity or availability of treatment resources. The presented risk score was developed as an easy-to-implement and observer-independent tool; therefore, we decided to include only demographic data and basic laboratory tests.

In line with other studies, we found that male sex was associated with a higher risk of death [11, 13, 21]. Older age was also identified as a predictor of death, as confirmed by other studies on COVID-19 related mortality [8, 9, 11–14]. Differences in age between our study cohorts may be related to the different time of recruitment. At the beginning of the pandemic, there was a tendency to admit more patients to the hospital because the health risks associated with COVID-19 was still unknown [22].

Laboratory abnormalities in patients with COVID-19 disease are common [23]. They can be related to the disease itself or comorbidities and related treatments that often aggravate the course of COVID-19, including all types of malignancies, cardiovascular disease, chronic kidney disease, chronic obstructive pulmonary disease, obesity (body mass index > 40 kg/m^2^), pregnancy, type 2 diabetes mellitus, sickle cell anemia, and medication use.

Anemia, leukocytosis, increased CRP and creatinine levels, low or high sodium and potassium levels as well as platelet count are associated with disturbed homeostasis and represent abnormalities that could be related to SARS-CoV-2 infection or concomitant diseases and their treatment. Mortality rates are higher in patients with COVID-19 and comorbidities [3, 4, 13]. It should be stressed, however, that comorbidities often present with abnormal laboratory findings even in patients without SARS-CoV-2 infection.

The association of selected demographic and laboratory features with outcome has been confirmed by many studies [3, 5, 7–9, 11, 13, 15]. Anemia in patients with SARS-CoV-2 infection could be present before the onset of infection due to chronic illness, especially malignancy. Anemia can develop in the course of SARS-CoV-2 infection due to inflammation including direct cytopathic injury of circulating erythrocytes and their bone marrow precursors, as well as damage due to hemolytic anemia, and/or thrombotic microangiopathy. An association between anemia and increased mortality was reported before [24] and is in line with our current findings.

Surprisingly, in our study, higher MCV levels were also associated with increased risk of mortality. This might be related to conditions that present with elevated MCV levels, such as hypothyroidism or vitamin B_12_ deficiency. However, further studies are needed to assess whether elevated MCV levels were also present before the infection. If not, this could indicate that SARS-CoV-2 infection might lead to changes in MCV levels [25]. In contrast to our finding, Djakpo et al. reported lower MCV levels in COVID-19 patients than in healthy participants [26]. However, MCV in non-survivors increased during hospitalization, resulting in the mean MCV values similar to those presented in our study.

The leukocyte count is an important parameter for predicting the severity of COVID-19 [27]. Huang et al. found that ICU patients with COVID-19 had a higher leukocyte count than non-ICU patients [28]. SARS-CoV-2 infection is primarily related to lymphopenia, which may reduce leukocyte count. However, this is followed by an increase in neutrophil count, leading to leukocytosis.

Increased CRP levels and leukocytosis are markers of infection severity and are related to increased mortality [29, 30]. Elevated CRP levels are present in numerous mortality risk scores used in patients with SARS-CoV-2 infection, but various studies used different cut-off points [8, 11, 14–16, 19]. In the current study, the cut-off point for CRP levels was at 51.5 mg/dL.

The significance of platelet count for outcome prediction has also been widely studied. In a previous report, platelet count did not differ between survivors and non-survivors. However, this might be explained by the presence of patients with both low and high platelet count in the studied group, which might have translated to normal mean count [31]. Furthermore, Lippi et. al. found that thrombocytopenia is related to a higher risk of adverse events during hospitalization [32].

Hyponatremia is a common finding in patients with pneumonia regardless of disease etiology [33]. However, it is more prevalent in patients with SARS-CoV-2 infection than in those with pneumonia related to other causes [34]. Our observation that hyponatremia is related to adverse outcomes in COVID-19 patients is concordant with other studies [35]. Hyponatremia is caused by various mechanisms, including the non-osmotic release of vasopressin induced by interleukin 6, the levels of which are increased in COVID-19 patients and inversely related to hyponatremia [36].

Hypernatremia, although rare, is also encountered in patients with SARS-CoV-2 infection and is related to higher mortality [37]. It may be caused by the loss of free water due to perspiration. It may also result from elevated renal sodium reabsorption due to increased angiotensin II activity following angiotensin-converting enzyme 2 receptor blockade by SARS-CoV-2 [38].

Hypokalemia is also commonly found in patients with COVID-19 pneumonia. Moreno-Perez et al. reported that hypokalemia is a sensitive biomarker of adverse COVID-19 progression [39]. In the present study, hypokalemia was a factor indicating poor outcome. Additionally, high potassium level was an unfavorable factor potentially associated with renal failure and treatment with potassium-sparing drugs, which may be an indicator of comorbidities.

Increased creatinine level was yet another predictor of adverse outcome in our patients, which is in line with the findings of other authors [3, 8, 37]. Yang et al. reported that almost 30% of COVID-19 patients with severe pneumonia showed increased creatinine levels [40]. High creatinine levels in patients with COVID-19 may be a sign of their concomitant diseases or may suggest that SARS-CoV-2 can induce kidney disease.

In our study, the accuracy and prognostic value of the covid-19 score was similar in the validation and developing cohorts. However, the cohorts differed in terms of age and sex distribution. This discrepancy may have resulted from different locations of the hospital: the developing cohort was hospitalized in a small town, while the validation cohort, in a larger city and a capital of one of the Polish administrative regions. Moreover, dialyzed patients with SARS-CoV-2 infection who received dialysis were admitted only to the hospital in Boleslawiec, where they received dialysis irrespectively of the presence of infection symptoms. The similar accuracy of the covid-19 score in these two cohorts confirms the significance of laboratory abnormalities.

The prediction of mortality in COVID-19 patients was also investigated by other authors, who used the Chinese protocol severity classification, pneumonia severity index (PSI), and Confusion-Urea-Respiratory Rate-Blood pressure-65 (CURB-65) in risk stratification and prognosis assessment [20]. The AUC of the Chinese protocol severity classification, PSI, and CURB-65 was 0.735, 0.951, and 0.912, which is in line with our results.

Studies based on results from a single laboratory facility showed lower predictive accuracy. For example, it was reported that lung ultrasound findings did not predict mortality [10]. The degree of lung involvement may be considered important but not critical to survival, which depends rather on systemic response to infection.

In our study, we did not exclude parameters that seemed to show a borderline association with mortality in the developing cohort. The significance of the assessed parameters may vary between different populations. This finding suggests that the analyzed parameters may have different significance depending on the population. However, further studies are needed to elucidate this issue. The use of the covid-19 score might be particularly useful during the pandemic, when the number of patients requiring hospitalization is particularly high and it is necessary to identify patients at increased risk of death. The value of our prediction model lies in its objectivity as well as the use of simple and widely available diagnostic tools. The model might be especially useful in a field hospital or a mobile medical unit. An initial assessment with the use of covid-19 score may facilitate decision-making regarding diagnostic and therapeutic strategies in patients with SARS-CoV-2 infection. In addition, the covid-19 score might help compare the effectiveness of treatment using different methods.

### Limitations

The main limitation of the study is the lack of clinical data related to comorbidities and treatments used. However, our aim was to develop a simple risk score that could be used in individual patients even by professionals that are less familiar with clinical assessment of COVID-19 cases.

Another limitation is the lack of data regarding the time from the onset of symptoms to hospital admission. Laboratory values might change during the infection, and the use of the scores obtained at different time points of the infection may be inadequate.

Finally, the inclusion criterion for the study was a positive result for COVID-19 during hospitalization. However, some patients may become infected during hospitalization, and laboratory abnormalities on admission may not necessarily indicate SARS-CoV-2 infection but also other conditions. Moreover, SARS-CoV-2 infection that develops during hospitalization may be asymptomatic. Additionally, in some cases, SARS-CoV-2 infection may not be diagnosed by the first smear test. Thus, it is possible that such patients were not included in the study cohort.

## Conclusions

The covid-19 score based on simple demographic characteristics and laboratory findings on admission is a reliable and valid tool for predicting in-hospital mortality in patients with SARS-CoV-2 infection.

## Data Availability

The datasets used and analyzed during the current study are available from the corresponding author on reasonable request.

## Abbreviations

AUC: Area under the receiver operating characteristic curve
COVID-19: Coronavirus disease 2019
CURB-65: Confusion-urea-respiratory rate-blood pressure-65
CRP: C-reactive protein
ICU: Intensive care unit
MCV: Mean corpuscular volume
ROC: Receiver operating curve
SARS-Cov-2: Severe acute respiratory syndrome coronavirus 2

## References

[1] Phelan AL, Katz R, Gostin LO (2020). The novel coronavirus originating in Wuhan, China: challenges for global health governance. JAMA.

[2] Tyrrell CSB, Mytton OT, Gentry SV, Thomas-Meyer M, Allen JLY, Narula AA (2020). Managing intensive care admissions when there are not enough beds during the COVID-19 pandemic: a systematic review. Thorax.

[3] Fumagalli C, Rozzini R, Vannini M, Coccia F, Cesaroni G, Mazzeo F (2020). Clinical risk score to predict in-hospital mortality in COVID-19 patients: a retrospective cohort study. BMJ Open.

[4] Halalau A, Imam Z, Karabon P, Mankuzhy N, Shaheen A, Tu J, Carpenter C (2021). External validation of a clinical risk score to predict hospital admission and in-hospital mortality in COVID-19 patients. Ann Med.

[5] Homayounieh F, Zhang EW, Babaei R, Mobin HK, Sharifian M, Mohseni I (2020). Clinical and imaging features predict mortality in COVID-19 infection in Iran. PLoS ONE.

[6] Berlin J (2020). Pandemic poses legal pitfalls: TMA seeks better liability shields. Tex Med.

[7] Almaghlouth NK, Davis MG, Davis MA, Anyiam FE, Guevara R, Antony SJ (2020). Risk factors for mortality among patients with SARS-CoV-2 infection: a longitudinal observational study. J Med Virol.

[8] Altschul DJ, Unda SR, Benton J, de la Garza RR, Cezayirli P, Mehler M, Eskandar EN (2020). A novel severity score to predict inpatient mortality in COVID-19 patients. Sci Rep.

[9] Bahl A, Van Baalen MN, Ortiz L, Chen NW, Todd C, Milad M, Yang A, Tang J, Nygren M, Qu L (2020). Early predictors of in-hospital mortality in patients with COVID-19 in a large American cohort. Intern Emerg Med.

[10] Bosso G, Allegorico E, Pagano A, Porta G, Serra C, Minerva V (2020). Lung ultrasound as diagnostic tool for SARS-CoV-2 infection. Intern Emerg Med.

[11] Chen Y, Linli Z, Lei Y, Yang Y, Liu Z, Xia Y (2020). Risk factors for mortality in critically ill patients with covid-19 in Huanggang, China: a single-centre multivariate pattern analysis. J Med Virol.

[12] Gupta RK, Marks M, Samuels THA, Luintel A, Rampling T, Chowdhury H (2020). Systematic evaluation and external validation of 22 prognostic models among hospitalised adults with COVID-19: an observational cohort study. Eur Respir J.

[13] Gupta S, Hayek SS, Wang W, Chan L, Mathews KS, Melamed ML (2020). Factors associated with death in critically Ill patients with coronavirus disease 2019 in the US. JAMA Intern Med.

[14] Hu C, Liu Z, Jiang Y, Shi O, Zhang X, Xu K (2020). Early prediction of mortality risk among patients with severe COVID-19, using machine learning. Int J Epidemiol.

[15] Knight SR, Ho A, Pius R, Buchan I, Carson G, Drake TM (2020). Risk stratification of patients admitted to hospital with COVID-19 using the ISARIC WHO Clinical Characterization Protocol: development and validation of the 4C Mortality Score. BMJ.

[16] Maguire D, Woods M, Richards C, Dolan R, Veitch JW, Sim WMJ (2020). Prognostic factors in patients admitted to an urban teaching hospital with COVID-19 infection. J Transl Med.

[17] Nguyen Y, Corre F, Honsel V, Curac S, Zarrouk V, Fantin B (2020). Applicability of the CURB-65 pneumonia severity score for outpatient treatment of COVID-19. J Infect.

[18] Quisi A, Alıcı G, Harbalıoğlu H, Genç Ö, Er F, Allahverdiyev S (2020). The CHA2DS2-VASc score and in-hospital mortality in patients with COVID-19: a multicenter retrospective cohort study. Turk Kardiyol Dern Ars.

[19] Torres-Macho J, Ryan P, Valencia J, Pérez-Butragueño M, Jiménez E, Fontán-Vela M (2020). The PANDEMYC score. An easily applicable and interpretable model for predicting mortality associated with COVID-19. J Clin Med.

[20] Wang X, Hu ZW, Hu Y, Cheng Y, Zhang H, Li HC (2020). Comparison of severity classification of Chinese protocol, pneumonia severity index and CURB-65 in risk stratification and prognostic assessment of coronavirus disease 2019]. Zhonghua Jie He He Hu Xi Za Zhi.

[21] Bienvenu LA, Noonan J, Wang X, Peter K (2020). Higher mortality of COVID-19 in males: sex differences in immune response and cardiovascular comorbidities. Cardiovasc Res.

[22] Bilaszewski L, Budrewicz K, Gogolewski G, Sycz K, Wolniakowski I, Madziarska K (2020). Hematology, C-reactive protein and procalcitonin in COVID-19 patients and historical pneumonia group Emerg. Med Serv.

[23] Lippi G, Plebani M (2020). Laboratory abnormalities in patients with COVID-2019 infection. Clin Chem Lab Med.

[24] Tao Z, Xu J, Chen W, Yang Z, Xu X, Liu L, Chen R (2020). Anaemia is associated with severe illness in COVID-19: a retrospective cohort study. J Med Virol.

[25] Gallagher PG (2017). Disorders of erythrocyte hydration. Blood.

[26] Djakpo DK, Wang Z, Zhang R, Chen X, Chen P, Antoine MMLK (2020). Blood routine test in mild and common, coronavirus (COVID-19) patients. Biosci Rep.

[27] Huang I, Pranata R (2020). Lymphopenia in severe coronavirus disease-2019 (COVID-19): systematic review and meta-analysis. J Intensive Care.

[28] Huang C, Wang Y, Li X, Ren L, Zhao J, Hu Y (2020). Clinical features of patients infected with 2019 novel coronavirus in Wuhan, China. Lancet.

[29] Bannaga AS, Tabuso M, Farrugia A, Chandrapalan S, Somal K, Lim VKA (2020). C-reactive protein and albumin association with mortality of hospitalised SARS-CoV-2 patients: a tertiary hospital experience. Clin Med (Lond).

[30] Ullah W, Thalambedu N, Haq S, Saeed R, Khanal S, Tariq S (2020). Predictability of CRP and D-Dimer levels for in-hospital outcomes and mortality of COVID-19. J Community Hosp Intern Med Perspect.

[31] Wool GD, Miller JL (2020). The impact of COVID-19 disease on platelets and coagulation. Pathobiology.

[32] Lippi G, Plebani M, Henry BM (2020). Thrombocytopenia is associated with severe coronavirus disease 2019 (COVID-19) infections: A meta-analysis. Clin Chim Acta.

[33] Tezcan ME, Dogan Gokce G, Sen N, Zorlutuna Kaymak N, Ozer RS (2020). Baseline electrolyte abnormalities would be related to poor prognosis in hospitalized coronavirus disease 2019 patients. New Microbes New Infect..

[34] Cuesta M, Thompson CJ (2016). The syndrome of inappropriate antidiuresis (SIAD). Best Pract Res Clin Endocrinol Metab.

[35] Królicka AL, Kruczkowska A, Krajewska M, Kusztal MA (2020). Hyponatremia in infectious diseases-a literature review. Int J Environ Res Public Health.

[36] Berni A, Malandrino D, Parenti G, Maggi M, Poggesi L, Peri A (2020). Hyponatremia, IL-6, and SARS-CoV-2 (COVID-19) infection: may all fit together?. J Endocrinol Invest.

[37] Asghar MS, Haider Kazmi SJ, Khan NA, Akram M, Hassan M, Rasheed U (2020). Poor prognostic biochemical markers predicting fatalities caused by COVID-19: a retrospective observational study from a developing country. Cureus.

[38] Zimmer MA, Zink AK, Weißer CW, Vogt U, Michelsen A, Priebe HJ, Mols G (2020). Hypernatremia-A manifestation of COVID-19: a case series. A A Pract.

[39] Moreno-P O, Leon-Ramirez JM, Fuertes-Kenneally L, Perdiguero M, Andres M, Garcia-Navarro M, COVID19-ALC research group (2020). Hypokalemia as a sensitive biomarker of disease severity and invasive mechanical ventilation requirement in COVID-19 pneumonia: a case series of 306 Mediterranean patients. Int J Infect Dis.

[40] Yang X, Yu Y, Xu J, Shu H, Xia J, Liu H (2020). Clinical course and outcomes of critically ill patients with SARS-CoV-2 pneumonia in Wuhan, China: a single-centered, retrospective, observational study. Lancet Respir Med.

